# Classification of multiple emotional states from facial expressions in head-fixed mice using a deep learning-based image analysis

**DOI:** 10.1371/journal.pone.0288930

**Published:** 2023-07-20

**Authors:** Yudai Tanaka, Takuto Nakata, Hiroshi Hibino, Masaaki Nishiyama, Daisuke Ino

**Affiliations:** 1 Department of Histology and Cell Biology, Graduate School of Medical Sciences, Kanazawa University, Kanazawa, Japan; 2 Department of Molecular and Cellular Pathology, Graduate School of Medical Sciences, Kanazawa University, Kanazawa, Japan; 3 Department of Pharmacology, Graduate School of Medicine, Osaka University, Kanazawa, Japan; Vellore Institute of Technology: VIT University, INDIA

## Abstract

Facial expressions are widely recognized as universal indicators of underlying internal states in most species of animals, thereby presenting as a non-invasive measure for assessing physical and mental conditions. Despite the advancement of artificial intelligence-assisted tools for automated analysis of voluminous facial expression data in human subjects, the corresponding tools for mice still remain limited so far. Considering that mice are the most prevalent model animals for studying human health and diseases, a comprehensive characterization of emotion-dependent patterns of facial expressions in mice could extend our knowledge on the basis of emotions and the related disorders. Here, we present a framework for the development of a deep learning-powered tool for classifying facial expressions in head-fixed mouse. We demonstrate that our machine vision was capable of accurately classifying three different emotional states from lateral facial images in head-fixed mouse. Moreover, we objectively determined how our classifier characterized the differences among the facial images through the use of an interpretation technique called Gradient-weighted Class Activation Mapping. Importantly, our machine vision presumably discerned the data by leveraging multiple facial features. Our approach is likely to facilitate the non-invasive decoding of a variety of emotions from facial images in head-fixed mice.

## Introduction

Most species in the animal kingdom are proposed to use a facial expression as a nonverbal mean to externally display emotions, as described in a literature by Charles Darwin in the 19th century [1]. To date, facial expressions have been well characterized in humans. Early studies by Paul Ekman [2] indicated the existence of the key facial expressions—happiness, sadness, anger, fear, surprise, disgust and neutral—, which can be discriminated based on distinct facial features. Through the combination of these key expressions, we humans are capable of conveying complex and diverse emotions to others during social interactions in daily life. In addition, recognizable changes in facial features appear in patients with disorders such as psychiatric symptoms, facial paralyses, and craniofacial anomalies [3–6]. Therefore, facial expressions are of interest to both basic and clinical researchers as a non-invasive method for diagnosing physical and/or mental conditions.

Then, what about in non-human animals? Various species, such as primates, canines, and rodents, exhibit facial expressions in response to emotional events [7–12]; therefore, the existence of facial expressions is likely in non-human animals. Considering that mice are one of the most commonly-used model animals for studying human health and disease, a quantitative assessment of facial expression in mice could advance our knowledge on emotions and related disorders. A pioneering work has established standardized metrics for categorizing painful facial expressions, known as the mouse grimace scale (MGS) [9]. Thus far, both a manual procedure and a machine learning-powered tool have been devised for scoring MGS [9,13]. However, these approaches do not enable the analysis of other emotional states. Reportedly, cutaneous sensory neurons underlying the production of positive emotions upon gentle touch are conserved from humans [14,15] to mice [16–19]. Therefore, in addition to aversive facial responses to painful stimulation, it is plausible that rodents, including rats [10] and mice [20], exhibit different facial expressions in response to positively-valenced stimuli, such as tickling and gentle touch, thus implying the existence of multiple facial expressions in these animals. Consequently, there is a need for standardized methodologies to unbiasedly classify diverse facial expressions, in order to gain a more profound understanding of emotion-dependent facial expression in mice. Recently, Dolensek and colleagues have reported a framework for a machine vision-assisted classification of multiple emotions from mouse facial images [11]. However, it is worth noting that their procedure represents an individual diagnosis, as their classifier has been trained using data from a single mouse and is solely applicable to that specific dataset. In the case of other species, including human [5,21] and primates [22], machine vision-assisted approaches, particularly those utilizing deep learning (DL)-mediated image analyses, have achieved automated facial recognition with a high accuracy using large datasets derived from multiple individuals. Therefore, if advanced tools like these were available for the classification of a variety of facial expressions in multiple mice, it would be possible to extend the analyses to a diverse range of experiments.

In this study, we present a DL-based technique for categorizing multiple emotions from lateral facial images of head-fixed mice. By using an optimized facial videography technique for head-fixed animals, which affords us to clearly capture the dynamics of key facial parts such as ear, eye, and mouth in a single focus, we efficiently acquired a dataset consisting of thousands of mouse facial images for three distinct conditions: the baseline state (neutral), the state elicited by tail-pinch stimulation (tail-pinch), and the state elicited by gentle abdomen brushing (brushing). After subjecting the raw images in the dataset to preprocessing such as background masking and image resizing, we devised a convolutional neural network (CNN)-based classifier for the three emotional states using SqueezeNet [23], a deep CNN architecture with high accuracy and lower computational costs. Our model exhibited remarkable sensitivity and specificity in the classification of untrained data. Furthermore, by combining the analysis using Gradient-weighted Class Activation Mapping (Grad-CAM) [24], a technique producing the focus of CNN-based machine visions, with subsequent manual image analysis, we identified the potential key features for the image classification. Overall, our methodology presented here offers a streamlined approach for classifying a diverse range of head-fixed mouse facial expressions.

## Materials and methods

### Animal surgery

**Fig 1:**
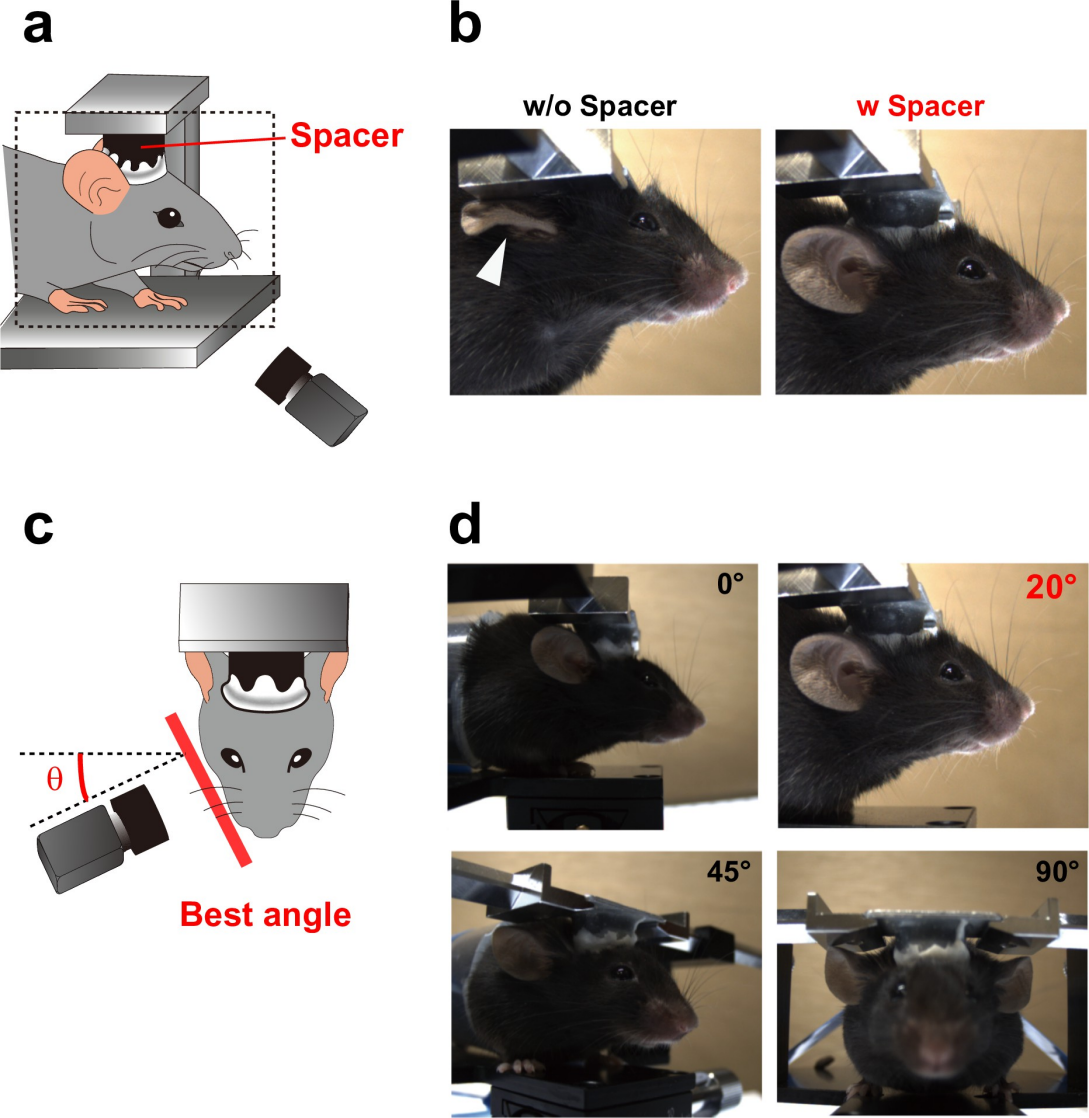
Optimization of facial videography in mice. **a**, Schematic illustrating facial videography in this study. **b**, Facial images captured either without- (left) or with a spacer (right). The spacer prevented ears from interference. **c**, Optimization of the angle for capturing facial expression in mice. **d**, Representative images captured at an angle of either 0°, 20°, 45° or 90° to the anterior-to-posterior axis.

C57BL/6J female mice aged 2−4 months were obtained from CLEA Japan (Tokyo, Japan). Surgery to attach a stainless headplate (CP-1, Narishige, Tokyo, Japan) with a spacer (Fig 1A) on the skull of the mouse using dental cement (Ketac Cem Easymix, 3M, Maplewood, MN, USA) was conducted under anesthesia of isoflurane. The depth of anesthesia was evaluated using the tail pinch method. Body temperature was maintained using a heating pad. Mice were returned to cages, and were raised in cages under a regular 12-h dark/light cycle with ab libitum access to food and water. Mice were recovered for at least one weak until the filming of facial images [11,25,26]. Total thirteen mice were used in this study.

### Ethical statement

All animal procedures were conducted following guidelines published by Ministry of Education, Culture, Sports, Science and Technology in Japan, and were approved by the ethics boards of Kanazawa University (No. 173903) and Osaka University (No. 03-020-010). All surgery was performed under isoflurane anesthesia, and all efforts were made to minimize suffering.

### Facial videography

To acquire the facial recordings, mice were held in a head fixation device (MAG-2, Narishige). The lower half of the body of the mouse was immobilized using a cylindrical tube crafted from a 50 mL conical tube. A uniformly colored wooden board was placed as a background screen. The faces of the mice were illuminated with white-colored light-emitting diodes on the floor, and were photographed from a 20° angle from the sides of the device (Fig 1C and 1D). To minimize the stress caused by immobilization, the mice were habituated on the device for a few ten minutes each day at least for a week before the experiments [11,25,27]. Facial images were acquired with a color CMOS camera (BFS-U3-23S3-C, FLIR Systems, Wilsonville, OR, USA) at a rate of 5 Hz controlled by a custom-made LabVIEW program (National Instruments, Austin, TX, USA), with minor changes from that used elsewhere [28].

### Stimulation protocols

Mice were subjected to either tail-pinch or brushing stimulus for 5 s, following a 60-s resting period. This procedure was repeated five times, with a 300-s interval between each iteration. For tail-pinch stimulation, the region approximately 1 cm from the proximal edge of the tail was gently grasped using ring forceps (outer diameter: 3 mm, inner diameter: 2.2 mm; 11103–09, FST, Foster City, CA, USA). As for the brushing stimulation, the abdomen was delicately brushed using a cotton ball affixed to a wooden stick (2 cm diameter; H050, PIP CO., LTD., Osaka, Japan). For acquisition of neutral faces, images were taken for 300 s without stimulation after the 60-s resting period. Given that mice can exhibit unfavorable responses to external stimuli such as loud sounds, we conducted the experiment in a quiet environment. To avoid the potential data biases, we applied only a single stimulus within a day, and tried the other stimulus on another day. If the head plate was detached from the mouse during the stimulation process, the corresponding video sequence was excluded from the analysis.

### Random image sampling

Images taken during 5 s-period after stimulation were used as sets of tail-pinch and brushing. In addition, images taken for 300 s without any stimulation were also included as neutral images. A total of 3099 “tail-pinch” images, 3999 “brushing” images, and 73598 “neutral” images were obtained. For the following analysis, the number of images in each group were adjusted to 3099 by random sampling.

### Image preprocessing

The initial step was to detect a mouse region in an input image (Fig 3B). We utilized a pre-trained model of Detectron2 for mouse detection, which is a PyTorch-based object detection library developed by Meta AI Research (New York City, NY, USA). To generate masked images, we combined the cropped mouse regions with a uniform background color (RGB = [200,167,122]). The processed images were subsequently resized to 227 × 227 pixels for the following network training.

### Network training

We constructed an AI model for classification of three different facial expressions using the preprocessed dataset. The calculation was conducted using MATLAB software. We employed hold-out method for the construction and evaluation of our AI-model. The dataset was randomly split into training, validation and test data, with a ratio 64:16:20, respectively. By using the training and validation data, we performed fine-tuning on SqueezeNet v1.1 architecture [23] with initial values of weights that had been trained using ImageNet data [29]. Data augmentation was conducted by applying random vertical flipping and random translation of up to 30 pixels in both horizontal and vertical directions. The parameters were configured as follows; optimization algorithm: stochastic gradient descent with momentum, mini-batch size: 512, loss function: cross-entropy error, learning rate: 3 x 10^−4^. The network was trained for 110 iterations with one validation for every ten iterations. Following the training of the model, a confusion matrix was created using the test data. Values of sensitivity and specificity were calculated as the percents of correct positive predictions divided by the total number of positives and correct negative predictions divided by the total number of negatives, respectively.

### Evaluation with Grad-CAM

Heatmaps were generated using Grad-CAM method [24] to visualize the regions of interest by CNN-based machine vision. The calculation was conducted using MATLAB software. In each dataset, the images with top five scores for classification were overlaid with the heatmaps (Fig 4).

### Post hoc image analysis

Ten geometrical parameters of facial parts in mice, including mouth opening, jaw angle, ear angle, ear eccentricity, ear perimeter, eye angle, eye eccentricity, eye perimeter, ear to eye angle, and ear to eye distance, were manually analyzed using Fiji [30]. The eccentricity and perimeter were calculated as the eccentricity and perimeter of the fitted ellipse, respectively. The angles of ear- and eye were measured as the angles formed by the major axis of the fitted ellipse with respect to the horizontal line. The jaw angle was measured as the angle formed by the line connecting the jaw and the mouth with the line connecting the mouth and the nose. The ear-to-eye angle measured derived as the angle formed by the line connecting the ear and the eye with the horizontal line. The ear-to-eye distance was measured as the distance between the centroids of the ear and the eye. We assigned a binary value of 1 for open mouths and 0 for closed mouths. The ten parameters were normalized to z-scores defined by (X-X_mean_)/X_SD_, where X_mean_ and X_SD_ are the mean, and standard deviation of the parameter values, respectively. Principal components were then calculated from the standardized measurements using scikit-learn for Python3. The principal components were then plotted in 3D coordinates.

## Results

### Optimization of facial image acquisition in mice

Rodents reportedly display various facial movements, including those of the ears, eyes, and mouth, during emotional responses [9–11,31]. Therefore, capturing the key parts clearly is critical to analyze the features during facial expressions. Head-fixation impedes the ability of mice to freely rotate in different directions and enables precise tracking of small facial features with high spatiotemporal resolution in mice [11,25,32]. We thus decided to capture fixed side-view facial images in mice that were immobilized through head-fixation. Initially, we attempted to acquire facial movies of head-fixed mice using a commercially available stereotaxic device; however, we discovered that the plate of the holding device often interfered with ear movement (Fig 1B left). To resolve this issue, we attached a cylindrical spacer between the mouse skull and the plate. With the presence of the spacer, the shape of the ear remained undistorted (Fig 1A and 1B). Subsequently, we optimized the angle that enables us to capture the entire facial features in mice (Fig 1C). Among the tested conditions, image acquisition at a 20° angle relative to the transverse direction of mice heads was found to be the most effective for capturing the key parts in focus (Fig 1D).

### Acquisition of neutral- and stimulation-evoked facial movies

**Fig 2:**
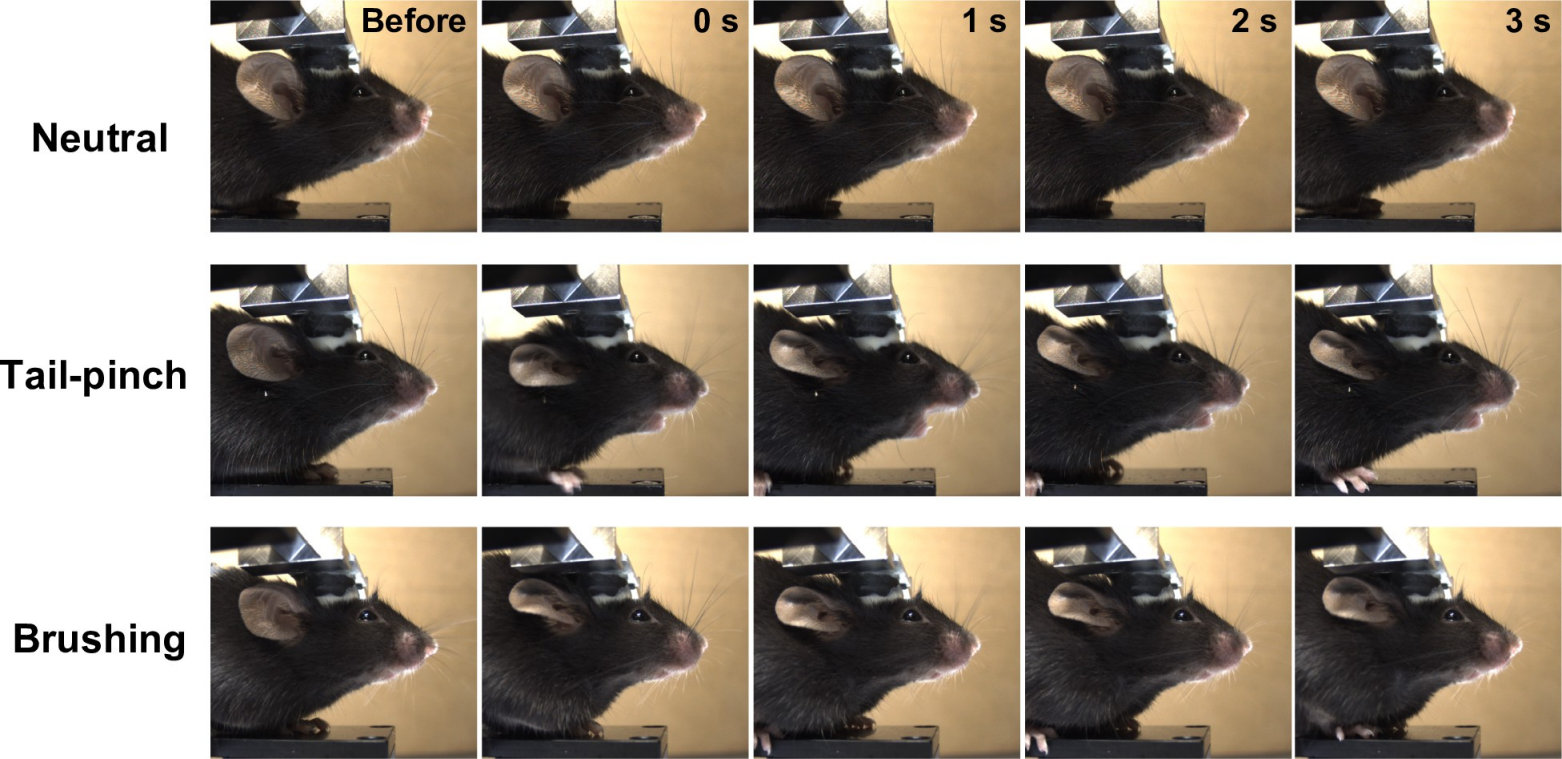
Facial dynamics of a mouse during three emotional states. Time-lapse images for facial dynamics during three emotional states (top: Neutral, middle: Tail-pinch, bottom: Brushing). Images were shown at 1-s intervals.

Using the optimized protocol above, we captured neutral- and stimulation-evoked facial movies in mice. To evoke negative- and positive emotions, we applied tail-pinch and brushing stimuli, respectively [10,11,33]. As shown in the representative images (Fig 2), mice reacted to both tail-pinch and brushing stimuli with robust movement in facial parts such as ear, mouth, and jaw, while they showed no detectable facial responses without stimulation. We repeatedly acquired the data using ten identical animals, and finally prepared the dataset containing 3099 images for each emotional state.

### Framework for our AI-based image classification

**Fig 3:**
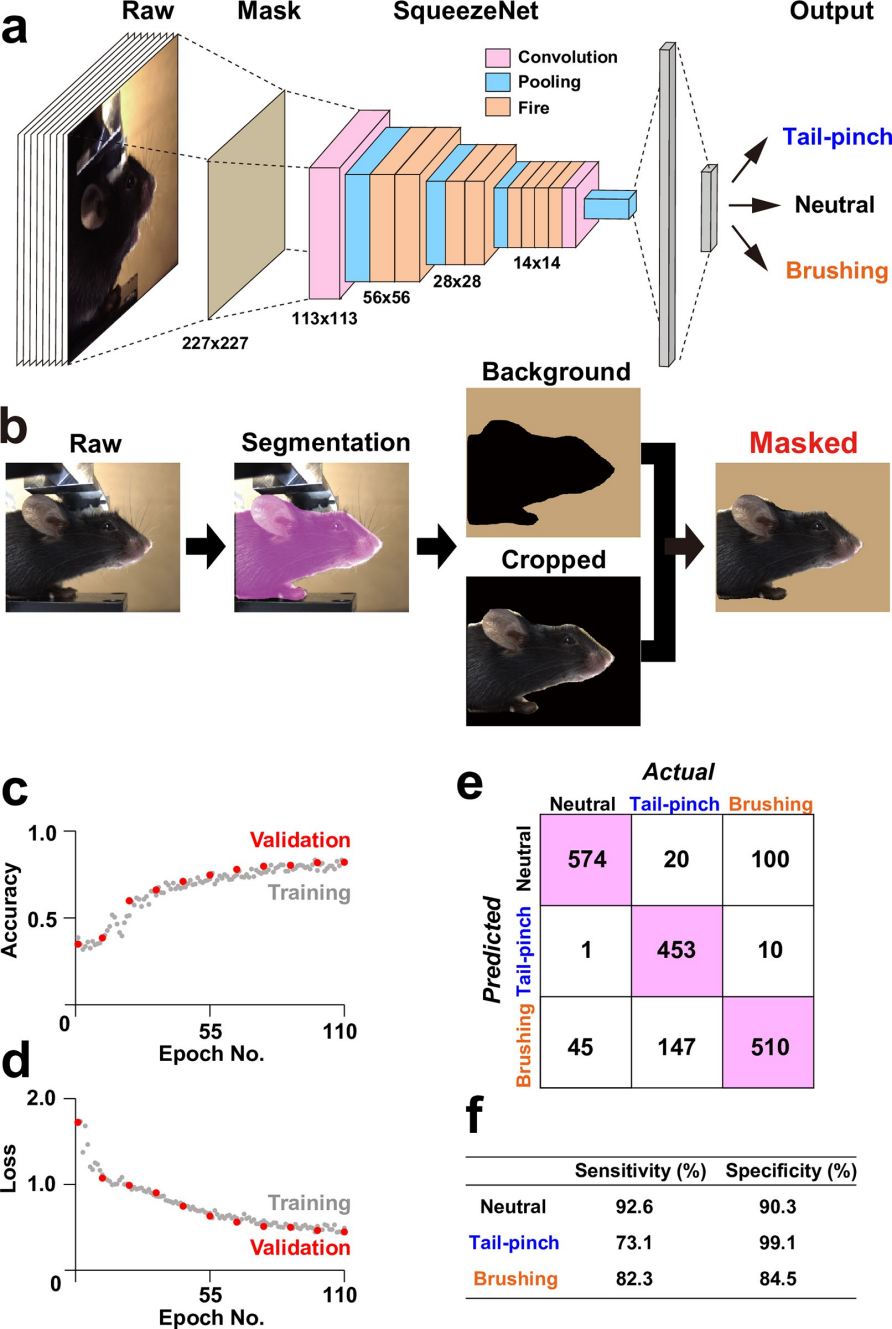
Framework of our AI model for facial recognition in mouse. **a**, Schematic of SqueezeNet-based our AI model for facial recognition in mouse. **b**, Schematic of creating masked images of mice. **c**−**d**, Learning curves of our model. **e**, Confusion matrix for classification of the test dataset. **f**, Summary of the values of sensitivity and specificity in **e**.

Although the patterns of facial movement were subtly distinct among three emotional states (neutral, tail-pinch, and brushing), intuitive recognition of the differences was difficult. Therefore, we decided to adopt an AI-based approach for an unbiased image classification. For this purpose, we employed SqueezeNet [23], an eighteen-layer deep CNN model with a high accuracy and fewer parameters, for image classification (Fig 3A). To remove the information of background objects such as a holding devise in raw images, we conducted masking of the mouse images using Detectron2, a CNN-based automated object detection tool, before the trainings in the network model (Fig 3A and 3B). By combining the cropped mouse images with the background colors, we generated input images for SqueezeNet. After resizing the images to 227 × 227 pixels, we constructed the model using training- and validation data. During 110-times iterations of trainings, parameters of learning curves improved well (Fig 3C and 3D). We evaluated the accuracy of the classifier by employing the test data, which had not been used for construction of the model (Fig 3E and 3F). The model exhibited accurate classification with notable sensitivities (92.6%, 73.1%, and 82.3% for neutral, tail-pinch, and brushing, respectively) and specificities (90.3%, 99.1%, and 84.3% for neutral, tail-pinch, and brushing, respectively). The results demonstrate the validity of our AI model to classify facial expressions during the three emotional states in mice.

### Mapping the facial features of each emotional state

**Fig 4:**
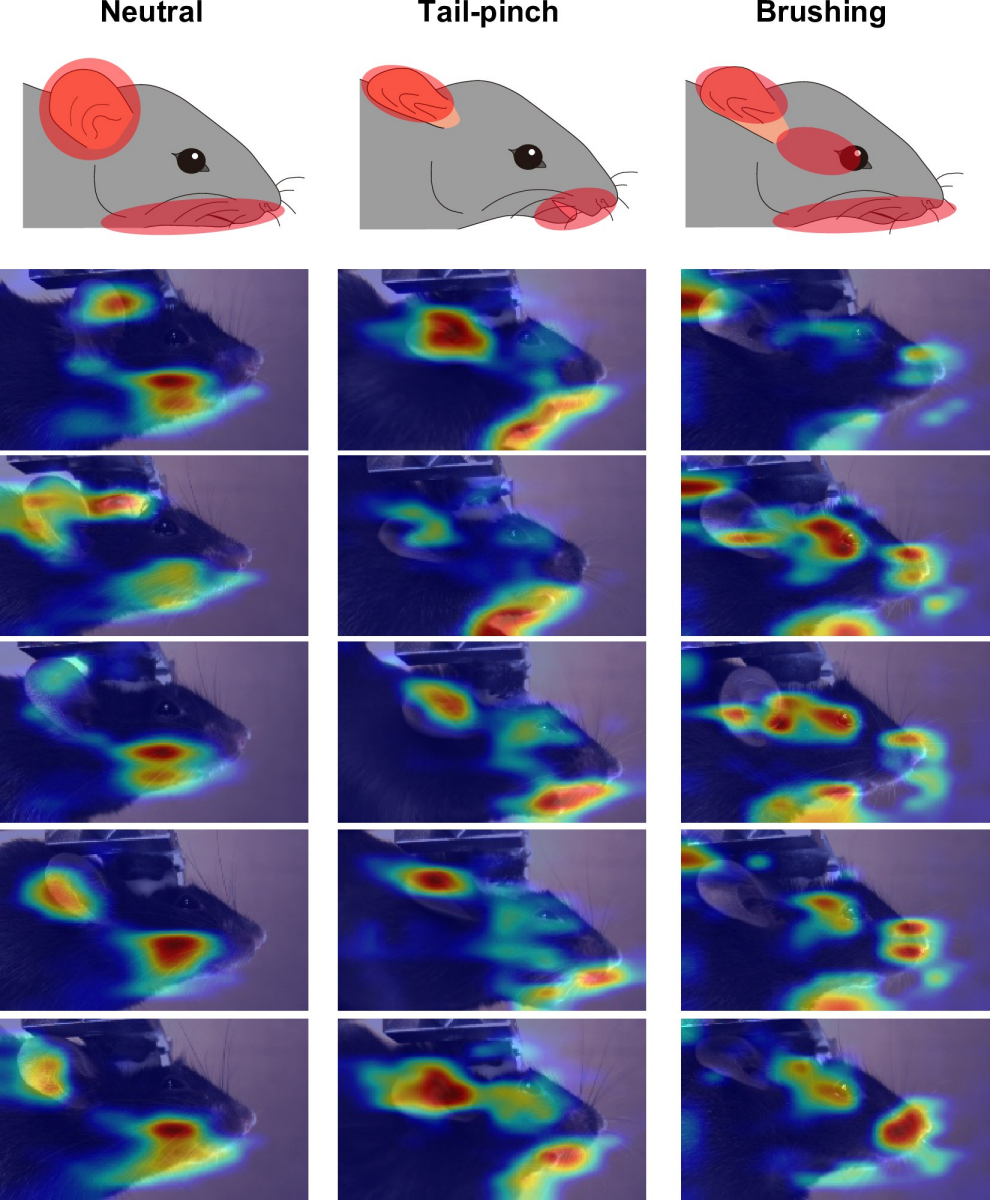
Mapping of the regions where our SqueezeNet based-model focused on. Representative images of Grad-CAM results in each emotional state. Images with top five classification scores were overlaid with heatmaps created by Grad-CAM analysis.

We next analyzed what features our AI model used for the image classification. To unbiasedly delineate the regions of interest of our AI, we employed Grad-CAM [24], which generates a heat map representing the visual rationale for decisions made by CNN-based models. We presented the representative heatmap images with five highest classification scores for each emotional state (Fig 4). The results indicate that strong signal appeared around ear, mouth, and jaw. Notably, our model appeared to focus on a flat ear and straight jawline in the neutral state, a crumpled ear and an open mouth in response to tail-pinch stimulation, and a crumpled ear, bent jawline, and possibly deformed eye in the context of brushing. The identified features near the ear and eye are consistent with the MGS [9] and those observed upon brushing stimuli [20]. Moreover, as discussed in a previous similar work [20], it is plausible that the features around the mouth (and potentially the jaw) may reflect the movements of key action units of the MGS, such as whisker, cheek, and nose [9].

### Verification of AI model interpretations by post hoc image analysis

**Fig 5:**
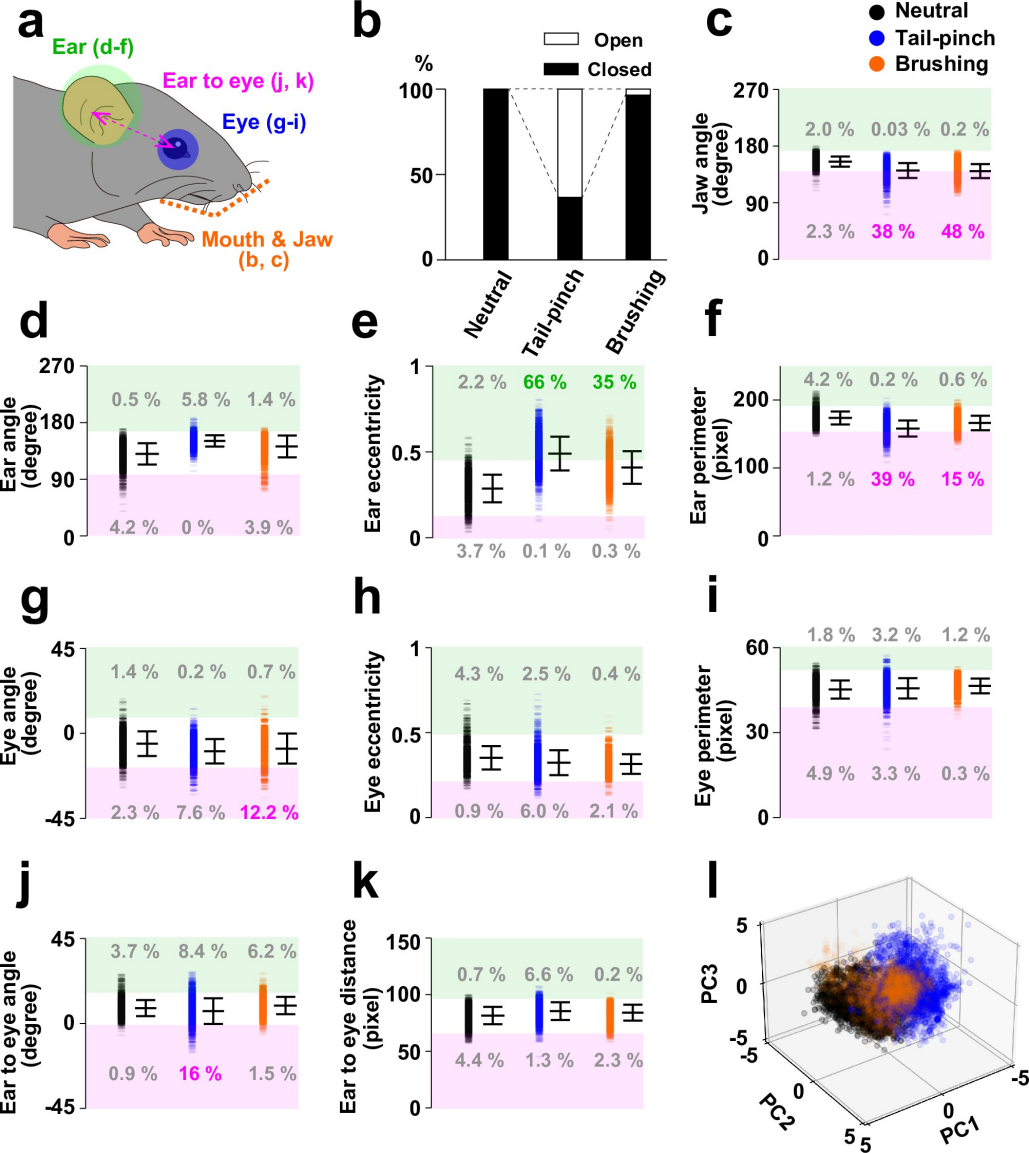
Post-hoc analyses of facial features. **a**, Schematic of analyzed regions in **b**−**k**. **b**, Fraction of open or closed mouth. **c-k**, Summary of parameters for the indicated features. Data are shown as mean ± SD. The upper and lower regions of mean ± 2SD intervals of “neutral” data are shaded with green and pink, respectively. The fraction of data (%) covered in the shaded regions are described. **l**, Scatter plot of the dataset after dimensionality reduction into three principal components.

Then, how did our AI model define the boundaries of three distinct groups by leveraging the discerned features from the Grad-CAM analysis? To gain insight into this question, we conducted quantitative analysis on the geometrical parameters for the featured facial parts (ear, mouth, and jaw) as well as eye, a critical parameter for rodent facial expression [9,10] (Fig 5A). We quantified ten parameters (mouth opening, jaw angle, ear angle, ear eccentricity, ear perimeter, eye angle, eye eccentricity, eye perimeter, ear to eye angle, ear to eye distance). As evident from the results of Grad-CAM analysis (Fig 4), we observed robust mouth opening in tail-pinch (Fig 5B), along with noticeable deviations of jaw angles (Fig 5C), ear eccentricities (Fig 5E), and ear perimeters (Fig 5F) in response both to tail-pinch and brushing stimuli. Moreover, we found discernible shifts in eye angle of brushing (Fig 5G), consistent with the results of Grad-CAM analysis. We also detected a slight change in the ear to eye angle of tail-pinch (Fig 5J), potentially reflecting the movement around the cheek, a key feature of the MGS [9]. However, with the exception of mouth opening—a distinctive and robust feature observed during tail-pinch stimulation—it was somewhat challenging to define clear boundaries for classifying the three groups using a single parameter, as the majority of data points exhibited substantial overlap in their distributions (Fig 5C–5K). Therefore, we conducted dimensionality reduction of the multivariate using principal component analysis. This analysis led to an apparent separation of the three emotional states (Fig 5L), suggesting that our AI-based model likely discriminated the data by leveraging multiple features of facial parts.

## Discussion

In this paper, we present a DL-assisted framework for the classification of multiple facial expressions in head-fixed mice. Utilizing the deep CNN architecture called SqueezeNet, we trained the model with a substantial number of images, enabling us to develop an AI-driven tool capable of accurately categorizing three distinct facial expressions (neutral, tail-pinch, and brushing) in mice. We demonstrated the efficacy of our model by successfully classifying untrained datasets obtained from various individual animals with notable sensitivity and specificity. By employing Grad-CAM analysis, we identified ear, eye, mouth, and jaw as potential key facial features for the classification by our model. Our manual analysis showed that distinctions in most of these key features were not always readily apparent, possibly due to trial-to-trial response variability of in vivo experiments, thereby rendering them less intuitively classifiable. Therefore, our DL-based approach will be powerful when automatically distinguishing subtle differences among multiple facial expressions.

Issues to be addressed are to expand the variation of facial classes. First, as proposed in a recent study [11], mice likely have several basic emotions similar to Ekman’s basic emotions in humans [2]. However, there are a fundamental question: how many patterns of emotion-dependent facial expression do mice possess? Since emotions are thought to originate in the brain, simultaneous recording of facial expression and brain activities, combining our videography with an electrophysiological measurement [34,35] or fluorescence imaging technique [36], could provide an answer. Second, elucidating the impact of stimulation intensities and types on facial expressions is also a critical concern. Obtaining training data encompassing various levels of stimulation strength and protocols will be important for advancing our model in the future. Because MGS is proposed to be a measure of spontaneous or non-evoked pain [9,37], the differences in facial patterns between the MGS and our tail-pinch data, which entailed acutely-evoked painful stimulation, may stem from the difference in the types of painful stimulation. Finally, while we employed C57/BL6J mice, one of the most prevalent mouse strains in laboratory experiments, it is crucial to consider the strain-specific attributes of mice, such as body size, fur color, and behavioral patterns [38]. Adapting the AI to classify the strain-dependent variations in facial expression among mice would confer notable advantages. Expanding our knowledge on the repertoires of facial expression in mice may also require the construction of databases containing a variety of facial expressions in mice, as is widely available for facial expression researches in human [39]. Considering that discernible movements around mouth and jaw upon either tail-pinch or brushing stimulation were observed, it is conceivable that mice may exhibit emotion-specific vocalization patterns as well [40,41]. Integration of our facial classifier with AI-assisted sound decoder will further enhance the precision of AI-assisted emotional evaluation in mice.

The past decade has witnessed remarkable advancements in artificial intelligence-assisted analysis for big data, owing to the emergence and rapid development of DL-based algorisms [42,43]. DL-assisted tools have made a huge impact on a wide range of research fields, including life and medical sciences. For behavioral researches in mice, such tools have enabled us to automatically capture and track the features of behaving mice, including body postures and vocalization patterns [44–46]. Our present work provides a new powerful tool for studying the association between facial expressions and emotions in head-fixed mice. Our AI-model will be extendable to the future work on the development of machine visions for freely-behaving mice, by training the model utilizing facial images captured from various angles. Refinements of the methodology, including the unification of object detection and classification into a single neural network, as well as the adaptation of the model to accommodate arbitrarily-sized images will also hold significance for broader applicability. Development of next-generation machine visions that can decode a wider variety of facial expressions more accurately may transform our ability to communicate nonverbally with laboratory animals.
